# Effect of Aspirin on Mitochondrial Dysfunction and Stress in the Pancreas and Heart of Goto-Kakizaki Diabetic Rats

**DOI:** 10.3390/life11090902

**Published:** 2021-08-30

**Authors:** Annie John, Layla Amiri, Jasmin Shafarin, Frank Christopher Howarth, Haider Raza

**Affiliations:** 1 Department of Biochemistry, College of Medicine and Health Sciences, UAE University, Al Ain P.O. Box 17666, United Arab Emirates; anniej@uaeu.ac.ae (A.J.); layla_i@hotmail.com (L.A.); jsalam@sharjah.ac.ae (J.S.); 2 Department of Physiology, College of Medicine and Health Sciences, UAE University, Al Ain P.O. Box 17666, United Arab Emirates; chris.howarth@uaeu.ac.ae

**Keywords:** GK rat pancreas and heart, type 2 diabetes, aspirin, mitochondrial functions, redox homeostasis, CYP 450s

## Abstract

Our previous study in Goto-Kakizaki (GK) type 2 diabetic rats provided significant evidence that aspirin treatment improves pancreatic β-cell function by reducing inflammatory responses and improving glucose tolerance. In the present study, we aimed to elucidate the mechanism of action of aspirin on the pathophysiology and progression of type 2 diabetic complications in the heart and pancreas of insulin-resistant GK rats. Aspirin treatment demonstrated a reduction in mitochondrial reactive oxygen species (ROS) production and lipid peroxidation, accompanied by improved redox homeostasis. Furthermore, the recovery of metabolic and mitochondrial functions, as well as cytochrome P450 enzyme activities, which were altered in the pancreas and heart of GK rats, were observed. Aspirin treatment brought the activity of CYP 2E1 to the control level in both tissues, whereas the CYP 3A4 level decreased only in the pancreas. This suggests the tissue-specific differential metabolism of substrates in these rats. The recovery of redox homeostasis could be the key target in the improvement of oxidative-stress-dependent alterations in mitochondrial functions which, in turn, facilitated improved energy metabolism in these tissues in the aspirin-treated GK rats. These results may have implications in determining the therapeutic use of aspirin, either alone or in combination with other clinically approved therapies, in insulin-resistant type 2 diabetes.

## 1. Introduction

The prevalence of diabetes has been steadily increasing all over the world, as a result of which it has become an epidemic in some countries. The global prevalence of diabetes in 2019 was estimated to be 463 million, rising to 578 million by 2030 and 700 million by 2045, with the prevalence being higher in urban than in rural areas and in high-income than in low-income countries [1]. It has also been recognized as one of the concomitant diseases which causes severe complications in patients with COVID-19 [2]. Diabetes is associated with multiple metabolic complications, characterized by intensive metabolic disturbances in different metabolic pathways, in most tissues resulting in high morbidity and mortality [3]. These complications are wide-ranging and are grouped as ‘microvascular’, which includes retinopathy, nephropathy and neuropathy, and the major ‘macrovascular’ complications include accelerated cardiovascular disease resulting in myocardial infarction and cerebrovascular disease manifesting as strokes [4,5]. Type 2 diabetes (T2D) was considered less complicated until a few years back, but this idea has changed since the life expectancy of patients has shortened and T2D has become a leading cause of death due to cardiovascular complications [3,6]. In addition to genetic, environmental and lifestyle risk factors, including inappropriate diet and visceral adiposity, the major causes of T2D are insulin secretory defects by the pancreatic islets and a decrease in its peripheral action. Obesity, inflammation, oxidative stress and mitochondrial dysfunction, as well as cardiometabolic syndrome, have been implicated as etiological and pathophysiological risk factors for T2D [7,8,9]. However, the exact mechanism of impairment and disease progression is still controversial. The Goto-Kakizaki (GK) rat, a commonly used insulin-resistant type 2 diabetic model for the study of non-obese T2D, is characterized by abnormalities in insulin secretion, glucose metabolism and chronic inflammation [10]. These animals, obtained through the repetitive breeding of glucose-intolerant Wistar rats, are genetic models of T2D and present the typical characteristics of T2D and its complications [11]. Growing evidence has linked T2D with chronic inflammation, thus making the GK rat a good animal model to study the pathophysiology of T2D and to investigate the effects of aspirin or other non-steroidal anti-inflammatory drugs (NSAIDs) [12,13,14].

Aspirin (acetylsalicylic acid, ASA), one of the most widely used NSAIDs, is a multifunctional drug, which affects different metabolic pathways in the body with various mechanisms [3]. The well-known mechanism of action of aspirin includes the inhibition of platelet function via the acetylation of COX-1, resulting in antithrombotic effects, and inhibition of prostaglandin production via the acetylation of COX-2, expressed by cytokines and other inflammatory stimuli [15]. It has been shown that low-dose aspirin (75–100 mg/day) was sufficient to inhibit COX-1, whereas intermediate doses (650 mg to 4 g/day) were required to inhibit COX-1 and COX-2 [16]. More recently, it has been suggested that the treatment of patients in the early stages of COVID-19 with low-dose aspirin (75–100 mg) is an important pharmacological strategy to prevent abnormal platelet aggregation, which causes thrombosis, leading to alterations in the lung and cardiovascular system [17]. For many years, aspirin has also been shown to attenuate insulin resistance and improve insulin secretion and signaling by decreasing nitric oxide-induced oxidative stress and reducing the levels of pro-inflammatory cytokines [18,19]. Researchers have shown that aspirin alone or in combination with hypolipidemic drugs potentiated their anti-oxidant effects and attenuated carbohydrate-related disturbances in diabetic rats [20,21]. Low-dose aspirin has been the cornerstone for the treatment of cardiovascular disease for several years, though some patients may be resistant to its effects [22,23,24,25]. Treatment of diabetic rats with high-dose aspirin has been shown to significantly inhibit heart glycogen accumulation, lower blood glucose and elevate glycolytic potential [3]. Our earlier studies on neonatal as well as adult diabetic rat models have shown increased oxidative and metabolic stress, impaired mitochondrial respiratory functions and alterations in redox metabolism in various tissues [26,27,28,29,30,31,32]. Our recent study on the same cohort of type 2 diabetic GK rats showed enhanced glucose tolerance and improved pancreatic beta-cell function by aspirin [33]. Insulin secretion from the pancreas and its transport and responses are well regulated by the physiological interactions of multiple tissues [34]. Studies have shown that hyperglycemia, dyslipidemia and hyperinsulinemia, which are hallmarks of diabetes, cause structural and functional changes and a pathological shift in circulating fuel levels and energy substrate utilization by central and peripheral tissues [35,36,37,38]. These changes include oxidative stress, metabolic and mitochondrial dysfunction, apoptosis and inflammation, causing systemic and tissue-specific metabolic defects. Since there is a close link between diabetes and cardiovascular complications, the main aim of our present study was to elucidate the metabolic and redox homeostasis, mitochondrial stress and dysfunction and drug metabolism and detoxification in the pancreas and heart of T2D rats, and to investigate the role of aspirin in recovering from the oxidative and metabolic stress in these tissues. We demonstrate that an improvement in redox homeostasis could be the key target, resulting in an improvement in oxidative stress-dependent alterations in mitochondrial functions which, in turn, facilitate better energy utilization in the pancreas and heart, resulting in improved glucose tolerance and insulin sensitivity, as shown in our previous study [33].

## 2. Materials and Methods

### 2.1. Chemicals

Aspirin (acetylsalicylic acid, ASA), reduced glutathione (GSH), oxidized glutathione (GSSG), 5,5′-dithio-bis (2-nitrobenzoic acid), 1-chloro 2,4-dinitrobenzene (CDNB), cumene hydroperoxide, dimethyl nitrosamine (DMNA), erythromycin, glutathione reductase, NADH, NADPH, coenzyme Q2, antimycin A, dodecyl maltoside, sodium succinate, cytochrome c, lucigenin and ATP Bioluminescent cell assay kits were purchased from Sigma-Aldrich Fine Chemicals (St. Louis, MO, USA). 2′,7′-dichlorofluorescein diacetate (DCFDA) was procured from Molecular Probes (Eugene, OR, USA). Kits for SOD and GDH were procured from Abcam (Cambridge, UK), and lipid peroxidation (LPO) kits were obtained from Oxis Int. Inc. (Portland, OR, USA).

### 2.2. Animals

Ten GK rats (male, five weeks old, weighing around 100–120 g) were procured from Taconic (Germantown, NY, USA). Control, male, non-diabetic rats (Wistar, n = 10) of similar age and weight were obtained from the Animal House Facility, College of Medicine and Health Sciences (Al Ain, U.A.E. University, United Arab Emirates) The animals had free access to food and water ad libitum and were maintained and handled as per the safe practices for animals as stipulated by the NIH, USA. Animal ethics approval (Protocol Ref#A1-13, dated 24 September 2013) was obtained from the Animal Ethics committee, College of Medicine and Health Sciences (U.A.E. University, United Arab Emirates).

### 2.3. Animal Treatment and Tissue Processing

At 4 months of age, the GK rats showed signs of hyperglycemia with a fasting blood glucose of 80–90 mg/dL and an average bodyweight of 319 g. The control Wistar rats, on the other hand, had a fasting blood glucose level of 50–60 mg/dL and an average bodyweight of 323 g. The animals were then divided into four subgroups: control, control + ASA, GK and GK + ASA, containing 5 animals each. The GK and Wistar control subgroups with ASA were injected intraperitoneally with 100 mg aspirin (ASA)/kg body weight/day for 5 weeks (aspirin was dissolved in normal saline and sodium hydroxide pellets were slowly added until the aspirin crystals dissolved completely and the pH of the solution was brought to pH 7.0). This experimental dose and these time points were selected based on the published reports in previous studies, using diabetic models including GK rats [39,40,41]. This dose and route of administration were previously shown to produce no gastric or renal toxicity in experimental animals [41,42,43,44].

At the end of 5 weeks, the animals were sacrificed via decapitation. The pancreas and heart from the animals were quickly excised and stored at −80 °C until further analysis. A portion of these tissues was homogenized (25% *w*/*v*) in H-medium (70 mM sucrose, 220 mM mannitol, 2.5 mM HEPES, 2 mM EDTA and 0.1 mM phenylmethylsulphonyl fluoride, pH 7.4) and used for isolating mitochondria, cytosol and microsome by means of differential centrifugation. The mitochondrial fraction was further purified via treatment with 75 μg/mg protein of digitonin and the resulting mitoplasts (mitochondria devoid of the outer membrane with the inner membrane intact and functional) were washed thoroughly to remove traces of digitonin and pelleted at 10,000 g. These mitochondrial preparations have been shown to contain less than 1% extra-mitochondrial cross-contamination, as described before [26]. The protein concentration in the different subcellular fractions was measured using the Bio-Rad reagent as described previously [30,33].

### 2.4. Measurement of Reactive Oxygen Species (ROS) Production and NADPH Oxidase (NOX) Activity

Reactive oxygen species (ROS) production in the pancreas and heart was evaluated using the DCFH-DA fluorescence method and the NADPH-dependent lucigenin-enhanced chemiluminescence method. ROS production in the mitochondrial extracts (mitoplasts) of control Wistar and GK rats treated with/without aspirin was measured using a cell-permeable probe, DCFH-DA (2′,7′-dichlorofluorescein diacetate) to detect the total peroxides since mitochondrial superoxides are easily converted to hydrogen peroxide by superoxide dismutase in the mitochondrial inner membrane [30]. Briefly, mitochondrial samples were incubated with 5 µM DCFH-DA for 30 min at 37 °C in potassium phosphate buffer, pH 7.4. The reaction was stopped using 100 µL of 0.1%Triton X-100 and read using a RF-5301 spectrofluorophotometer (Schimadzu Corporation, Kyoto, Japan) at an excitation wavelength of 488 nm and an emission wavelength of 525 nm. ROS production was also measured as NADPH oxidase (NOX) activity using the lucigenin-enhanced chemiluminescence method. Briefly, tissue homogenates were treated with lucigenin, in the presence of NADPH in potassium phosphate buffer, pH 7.4, and chemiluminescence was instantly measured using the Turner Designs TD-20/20 luminometer (Turner Designs, Sunnyvale, CA, USA).

Microsomal lipid peroxidation was measured using the LPO-586^TM^ assay kit according to the vendor’s recommended protocol and the concentration of malondialdehyde was calculated from the standard curve. SOD activity was measured using the SOD kit, as the percentage reduction of NBT to NBT-diformazan according to the manufacturer’s protocol. The percentage reduction in formazan formation was used as a measure of SOD activity.

### 2.5. Measurement of GSH and GSH Metabolism

The concentration of GSH, the most important cellular antioxidant, was measured in the cytosol and mitochondria using the NADPH-dependent glutathione reductase catalyzed conversion of oxidized glutathione to reduced glutathione, by means of the enzymatic recycling method of Tietze [45]. Glutathione S-transferase (GST) activity using CDNB, glutathione peroxidase (GSH-Px) activity using cumene hydroperoxide and glutathione reductase activity using GSSG/NADPH as respective substrates were measured using standard protocols on the UV-1800 spectrophotometer (Schimadzu Corporation, Kyoto, Japan) [46,47,48].

### 2.6. Measurement of Cytochrome P450-Dependent 2E1 and 3A4 Activities

Cytochrome P450-dependent enzymes play an important role in metabolizing and detoxifying many endogenous compounds and a wide variety of xenobiotics and drugs. CYP 2E1-dependent N-demethylase activity was measured in the microsomal fraction of the pancreas and heart of control Wistar and GK rats treated with/without aspirin, using dimethyl nitrosamine (DMNA) as the substrate in the presence of NADPH [49]. Similarly, the catalytic activity of CYP 3A4 enzymes was measured in the microsomal fractions using erythromycin as a substrate [50].

### 2.7. Measurement of Mitochondrial Respiratory Complexes and ATP Level

Freshly isolated pancreas and heart mitochondrial fractions (5 μg protein) from control Wistar and GK diabetic rats treated with/without aspirin were suspended in 20mM KPi buffer, pH 7.4, in the presence of lauryl maltoside (0.2%). NADH-ubiquinone oxidoreductase (Complex I), succinate-cytochrome reductase (Complex II + III) and cytochrome c oxidase (Complex IV) activities were measured using their respective substrates, coenzyme Q2, succinate and reduced cytochrome c, according to the method of Birch-Machin and Turnbull [51], as described previously [26,27,28,29]. The ATP content in the mitochondrial fractions of pancreas and heart was measured using the ATP Bioluminescent kit (Sigma, St. Louis, MO, USA) as per the manufacturer’s instructions and the luminescence was read using a TD-20/20 luminometer (Turner Designs, Sunnyvale, CA, USA).

### 2.8. Measurement of Glutamate Dehydrogenase Activity

Glutamate dehydrogenase (GDH), a mitochondrial enzyme, plays an important role in controlling insulin secretion. GDH activity was measured using the GDH kit (Abcam, Cambridge, UK) as per the vendor’s protocol. Briefly, the mitochondrial samples from the pancreas and heart of control Wistar and GK diabetic rats were incubated in a reaction buffer, containing glutamate and the NADH produced was measured spectrophotometrically using the UV-1800 spectrophotometer (Schimadzu Corporation, Kyoto, Japan) at 450 nm using a standard curve.

### 2.9. Statistical Analysis

Values shown are expressed as mean ± SD of three individual experiments. The statistical significance of the data was assessed using SPSS software (version 23) by means of an analysis of variance, followed by LSD post hoc analysis. *p*-Values ≤ 0.05 were considered statistically significant.

## 3. Results

### 3.1. Sub-Cellular Oxidative Stress in the Pancreas and Heart of Diabetic Rats: Protection by Aspirin

**Figure 1:**
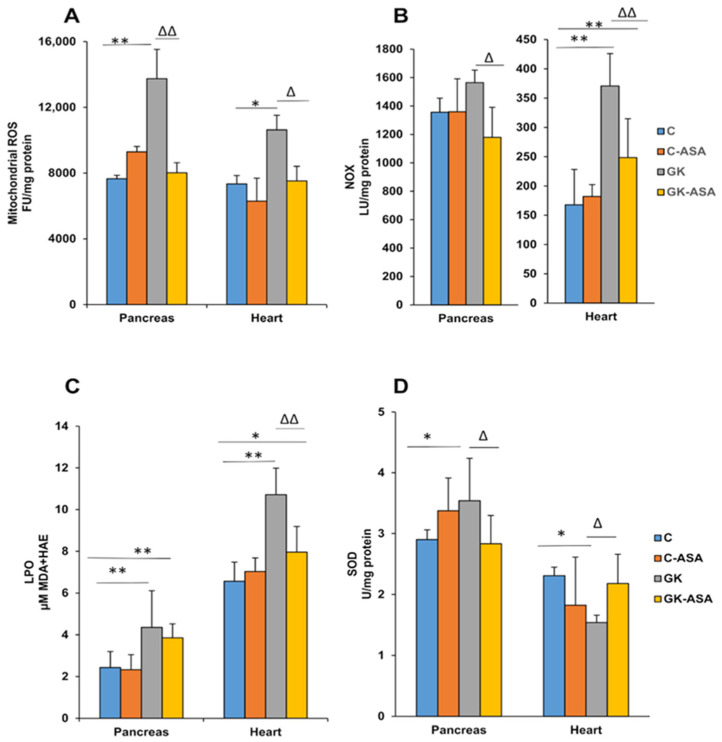
Effects of aspirin (ASA) treatment on mitochondrial ROS and membrane-bound NOX activity, lipid peroxidation and SOD levels in the pancreas and heart of control Wistar and GK rats (n = 5). Mitochondrial ROS production (**A**) in the pancreas and heart of control Wistar and GK rats was measured using DCFDA as a probe. Total homogenates from the tissues of these animals were analyzed for membrane-bound NOX production (**B**) using the lucigenin-enhanced chemiluminescence method, as described in the Materials and Methods. NADPH-dependent LPO (**C**) was measured in the pancreas and heart of control Wistar and GK rats using the LPO-586™ assay kit from Oxis Int. Inc. (Portland, OR, USA) and SOD levels (**D**) were measured using the SOD kit from Abcam (Cambridge, UK) as per the manufacturer’s protocol. Results are expressed as mean ± SD. from three independent experiments and asterisks indicate a significant difference. * *p* < 0.05 compared to control, ** *p* < 0.001 compared to control, ∆ *p* < 0.05 compared to GK, ∆∆ *p* < 0.001 compared to GK animals.

A significant increase in ROS production was observed in the mitochondrial extracts of the pancreas (~80%) and heart (~45%) of GK rats, which was reduced almost to control levels after aspirin treatment (Figure 1A). Control Wistar rats treated with aspirin showed no significant ROS formation. Similarly, membrane-bound NADPH oxidase increased significantly only in the heart of GK rats, whereas the pancreas showed only a mild increase (Figure 1B). Aspirin treatment markedly reduced the production of ROS in the hearts of GK rats, though a moderate reduction was seen in the pancreas. This could suggest differential sources of ROS production in the pancreas and heart in GK rats. Microsomal lipid peroxidation was also significantly increased (60–80%) in both the pancreas and heart of GK rats (Figure 1C). This could be due to increased peroxide formation, suggestive of increased oxidative stress in diabetic rat tissues. Aspirin treatment markedly reduced the levels in both tissues in GK rats, though the values were still above the control levels. A significant increase in pancreatic SOD and a decrease in heart SOD levels was observed in GK rats (Figure 1D). Aspirin treatment again brought the values close to the control values. No appreciable difference was observed in the control rats treated with aspirin.

### 3.2. Alterations in GSH-Dependent Redox Metabolism in Pancreas and Heart Improved by Aspirin Treatment

**Figure 2:**
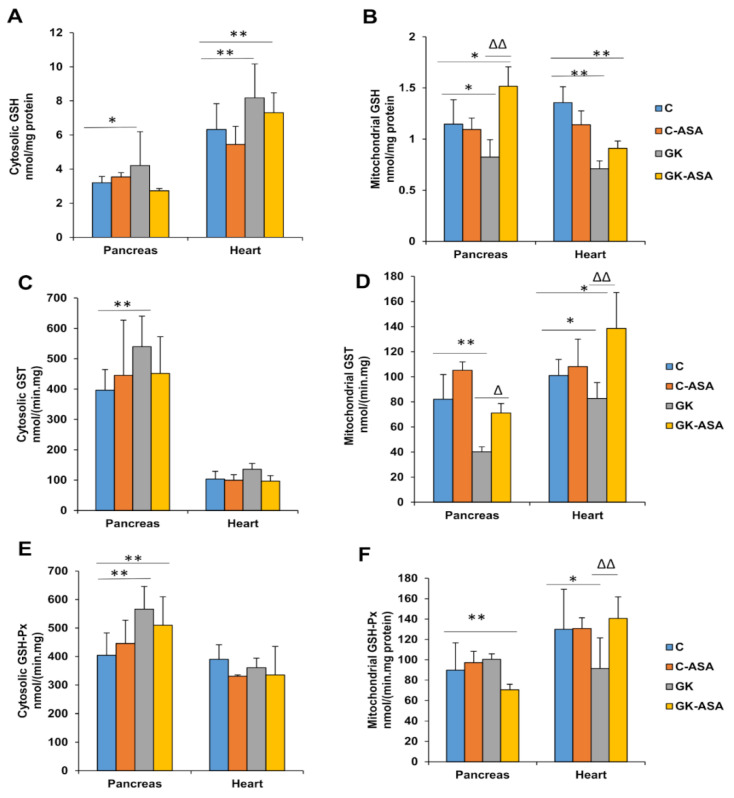

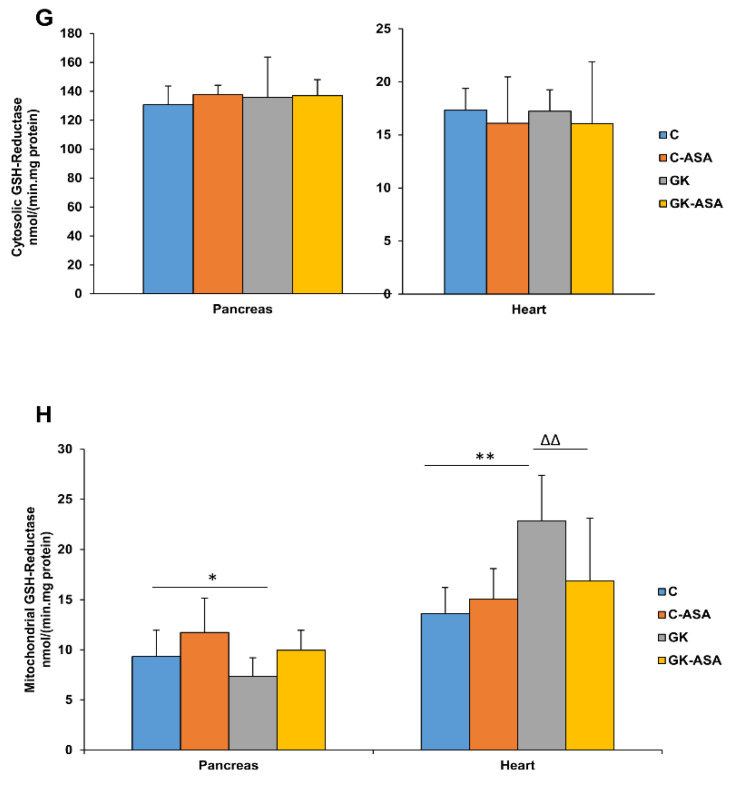
Glutathione levels and metabolism in the pancreas and heart of control Wistar and GK rats treated with aspirin (ASA) (n = 5). GSH concentrations in the pancreas and heart of control Wistar and GK rats were measured in the cytosol (**A**) and mitochondria (**B**) using the enzymatic recycling method of Griffith, as described in the Materials and Methods. GST activity, using CDNB as a substrate, was measured in the cytosol (**C**) and mitochondria (**D**). GSH-Px activity in the pancreas and heart of control Wistar and GK rats was measured in the cytosol (**E**) and mitochondria (**F**) using cumene hydroperoxide as a substrate and GSH-reductase activity was measured in the cytosol (**G**) and mitochondria (**H**) using GSSG/NADPH as a substrate. Values are means ± SD from three independent experiments. Asterisks indicate a significant difference. * *p* < 0.05 compared to control, ** *p* < 0.001 compared to control, ∆ *p* < 0.05 compared to GK, ∆∆ *p* < 0.001 compared to GK animals.

A significant increase (30–40%) in the cytosolic GSH levels and a significant decrease in mitochondrial GSH were observed in the pancreas and heart of GK animals, which came close to normal levels after aspirin treatment (Figure 2A,B). GSH-conjugating activity by GSH S-transferase (GST) in the cytosolic fraction showed a significant increase in the pancreas, whereas only a mild increase was observed in the heart (Figure 2C) of GK rats. However, the mitochondrial fractions showed a significant decrease in GST activity in the pancreas and heart of GK rats and aspirin treatment increased the activity, though more significantly in the heart (Figure 2D). This could be due to the increased conjugation of the GSH due to the increased mitochondrial ROS production in the heart. These results suggest the differential response in maintaining the GSH pool and its conjugating activity in the pancreas and heart of diabetic GK animals.

Increased GSH-Px activity in the pancreas was observed in the cytosolic fraction of GK rats, which was marginally reduced by aspirin treatment (Figure 2E). The heart, however, showed no significant alterations in the cytosol, whereas a mild decrease was observed in the mitochondria, which came back to control levels after aspirin treatment (Figure 2F). This may suggest differential oxidative and redox responses in the heart and pancreas of diabetic rats.

Increased GSH-reductase activity was observed in the mitochondrial fraction of the GK heart compared with the mild decrease in the pancreas (Figure 2H). This again shows the differential response in maintaining the GSH pool in the pancreas and heart of GK animals. However, cytosol did not show any alterations in both the pancreas and heart (Figure 2G) of GK rats.

### 3.3. Alterations in Cytochrome P450 Enzyme Activities by Aspirin Treatment

**Figure 3:**
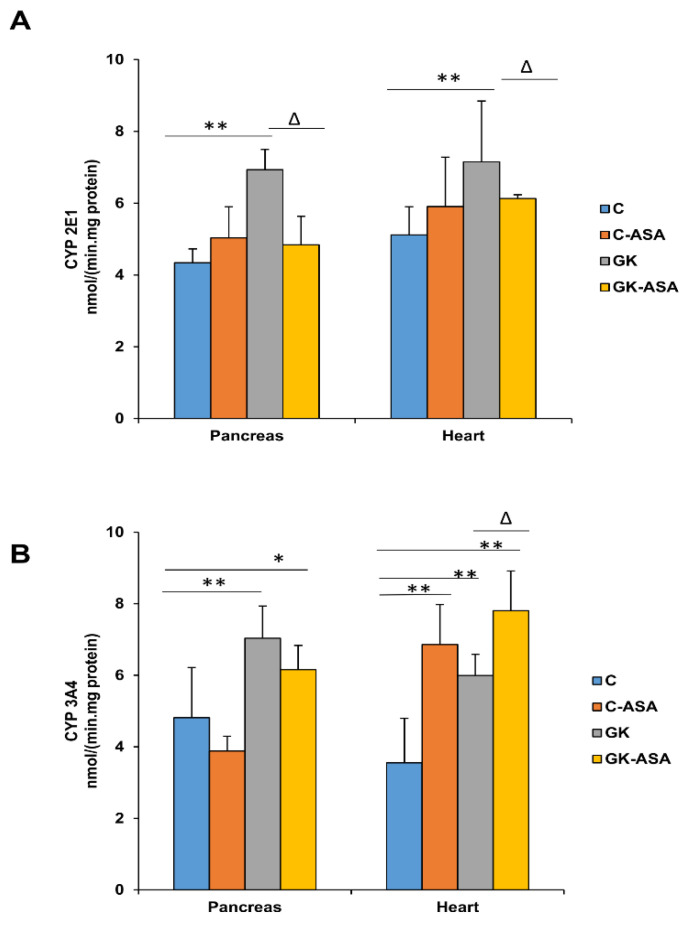
CYP 450 levels in the pancreas and heart of control Wistar and GK rats treated with aspirin (ASA) (n = 5). CYP 2E1 (**A**) and CYP 3A4 (**B**) activities in the pancreas and heart microsomal fractions were measured using standard substrates, as described in the Materials and Methods. Results are expressed as mean + SD from three independent experiments and asterisks indicate a significant difference. * *p* < 0.05 compared to control, ** *p* < 0.001 compared to control, ∆ *p* < 0.05 compared to GK rats.

A significant increase (60%) in CYP 2E1 activity in the pancreas and a 35% increase in the heart (Figure 3A) of GK rats was observed. This increase in activity could be due to increased oxidative stress. Aspirin treatment brought the activity to the control level in both the tissues. Similarly, CYP 3A4 activity was also significantly increased in both the pancreas and heart (Figure 3B) of GK rats. However, aspirin treatment could not bring the activity back to control levels in both the tissues. The activity increased further after aspirin treatment in the heart tissue. This could be due to the tissue-specific differential expression of CYP isoenzymes and their involvement in aspirin metabolism.

### 3.4. Alterations in Mitochondrial Bioenergetics in Diabetic Rats: Protection by Aspirin

**Figure 4:**
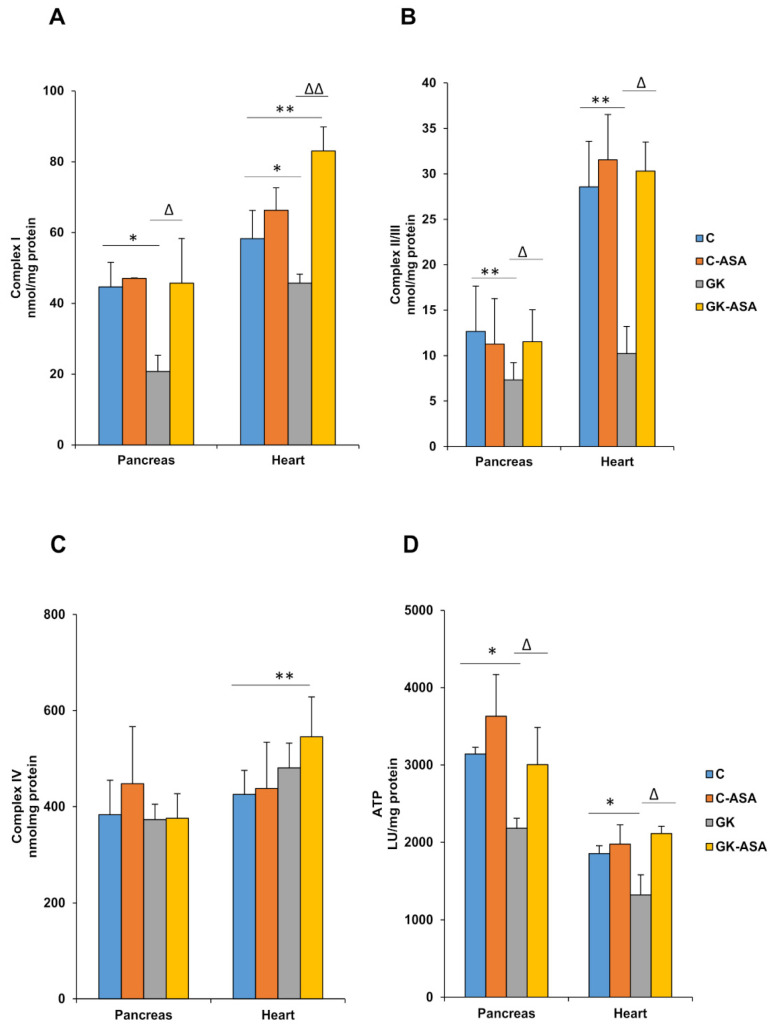
Effect of aspirin (ASA) on the activities of mitochondrial complexes and ATP levels in the pancreas and heart of control Wistar and GK rats (n = 5). Mitochondrial respiratory enzyme Complexes I (**A**), II/III (**B**) and IV (**C**) activities were measured in freshly prepared mitochondrial fractions from the pancreas and heart of control Wistar and GK rats using enzyme-specific substrates, as described in the Materials and Methods. The ATP content (**D**) in the rat tissues was determined using the ATP Bioluminescent cell assay kit (Sigma, St. Louis, MO, USA). Results are expressed as mean + SD from three independent experiments and asterisks indicate a significant difference. * *p* < 0.05 compared to control, ** *p* < 0.001 compared to control, ∆ *p* < 0.05 compared to GK, ∆∆ *p* < 0.001 compared to GK rats.

Respiratory Complex I activity was markedly reduced in both pancreas and heart mitochondria of GK rats (Figure 4A). Treatment with aspirin increased the activity to almost control levels. Similarly, Complex II/III activity was also significantly reduced in both the tissues of GK rats (Figure 4B). Treatment with aspirin again significantly increased the activity to control levels in both the pancreas, as well as in the heart of GK rats. However, no significant alterations in respiratory Complex IV activity were found in either the pancreas or heart (Figure 4C). This may suggest a possible resilience of the mitochondrial bioenergetic function to sustain ATP production and maintain insulin secretion. Mitochondrial ATP levels were markedly reduced in the pancreas and heart of GK animals (Figure 4D) though aspirin treatment brought the values to similar levels as those of control rats. This shows the beneficial effects of aspirin treatment by increasing energy utilization and ATP production.

### 3.5. Aspirin Improves Glutamate Dehydrogenase Activity in Pancreas and Heart of Diabetic Rats

**Figure 5:**
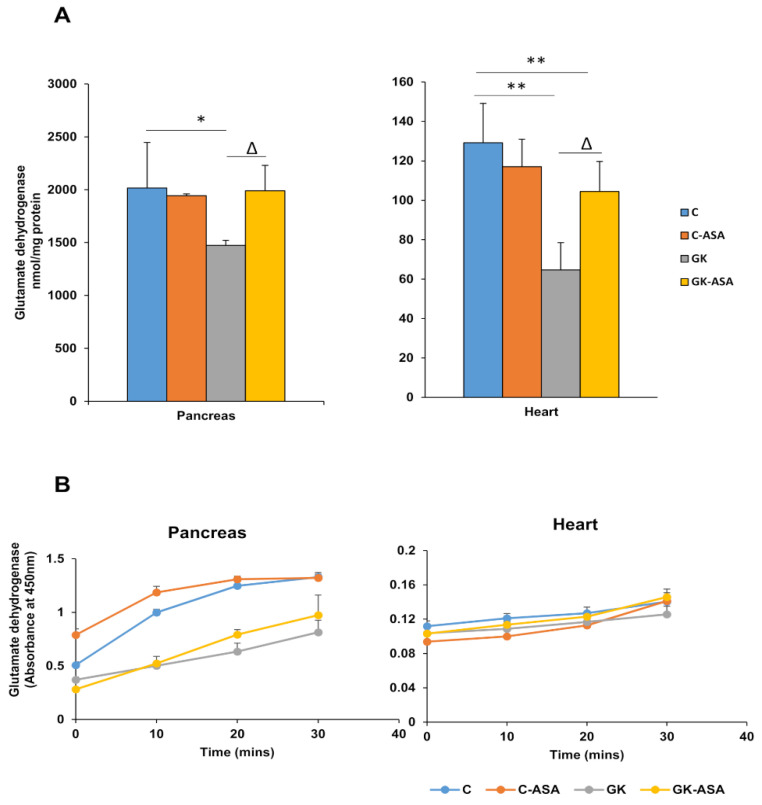
Effect of aspirin (ASA) on glutamate dehydrogenase activities in the pancreas and heart of control Wistar and GK rats (n = 5). Glutamate dehydrogenase activity was measured in the pancreas and heart of control Wistar and GK rats using the kit protocol, as described in the Materials and Methods, and the NADH produced after 30 min of reaction was determined using a standard curve (**A**). A time course reaction with absorbance values at 450 nm over 30 min at 10 min intervals is shown in (**B**). Values are means ± SD from three independent experiments and asterisks indicate a significant difference. * *p* < 0.05 compared to control, ** *p* < 0.001 compared to control, ∆ *p* < 0.05 compared to GK rats.

The activity of the mitochondrial enzyme glutamate dehydrogenase (GDH) was significantly reduced in both the tissues of GK rats (Figure 5A). However, aspirin treatment improved the levels significantly. No change in activity was observed in the control rats treated with aspirin. These results imply improved energy utilization in the pancreas and heart of GK diabetic animals after aspirin treatment.

## 4. Discussion

T2D is a multi-factorial metabolic disorder, characterized by defects in insulin secretion and/or responses, increased inflammation, compromised energy metabolism and cardiovascular abnormalities. However, the precise mechanism of disease progression and complications is still not clear. The GK rat, a spontaneous non-obese animal model of T2D, is a good animal model to study the pathophysiology of T2D and to study the effects of aspirin and other NSAIDs [12,13,52]. Researchers have shown that the influence of aspirin and salicylates is multifactorial and involves beneficial and deleterious effects, depending on the species and experimental model studied [53].

Our previous study on the same cohort of animals demonstrated that treatment with 100 mg/kg body weight aspirin for 5 weeks had marginally lowered the body weight in control Wistar as well as GK diabetic rats [33]. Blood glucose levels in GK rats after aspirin treatment were also shown to have moderately decreased, which could be due to more efficient glucose uptake after aspirin treatment. Improved glucose tolerance accompanied by an enhancement of pancreatic endocrine function and an enhanced insulin response were also observed after aspirin treatment [33]. Immunofluorescence studies showed that aspirin treatment improved the integrity and morphology of pancreatic islets, which were degenerated in the GK rats. Furthermore, serum enzyme analysis, including liver and renal function tests, showed no signs of organ toxicity, though lipid profiles showed a significant increase in cholesterol and total lipoproteins. An improvement of dyslipidemia was also reported after aspirin treatment in these animals [33]. The main aim of our present study was to investigate the effects of aspirin on energy and glutathione-dependent redox metabolism, oxidative stress and mitochondrial dysfunction in the pancreas and heart of GK rats. We have demonstrated an increase in energy metabolism and mitochondrial function after aspirin treatment, as well as alterations in the activities of cytochrome P450 and GSH-conjugating drug-metabolizing enzymes.

Oxidative stress plays a key role in the pathophysiology of insulin resistance and diabetes, via various molecular mechanisms, including beta cell dysfunction, inflammatory responses and mitochondrial dysfunction [54,55]. Researchers have also shown that oxidative stress is involved in the pathophysiology of diabetic cardiomyopathy and heart failure [7]. Our present study also exhibited increased mitochondrial ROS production and NADPH oxidase (NOX) activity that generated ROS, and increased LPO in the pancreas and heart of GK diabetic rats. Studies have shown increased NOX activity to be associated with impaired calcium signaling and hyperglycemia-induced cardiomyocyte apoptosis and mitochondrial dysfunction, as well as the regulation of insulin secretion in pancreatic cells [36,37,56]. In addition to hyperglycemia, dyslipidemia has also been associated with NOX activation [35]. In our study, aspirin treatment attenuated oxidative stress by decreasing ROS production and NOX activity in GK rats. Inhibition of NADPH oxidase has been shown to alleviate oxidative stress and mitochondrial dysfunction [57,58]. Inhibition of NADPH oxidase also conferred protection to beta cells against cytokines or free fatty acids, which is also a hallmark of diabetes [59]. A decrease in SOD activity was observed in the heart of these animals, suggesting increased oxidative stress, which could be due to the decreased level of antioxidants in cardiac tissue. However, a slight increase in SOD activity was observed in the pancreas, indicating that the increased mitochondrial ROS production in the pancreas could be due to superoxide anions. Excessive superoxide production, either by NOX activity or mitochondrial respiration, can accelerate the deleterious metabolic complications in diabetes. Aspirin treatment brought the values close to control levels. Studies have shown that targeting ROS production could be an important pharmacological therapy for preventing hyperglycemia-induced cardiac dysfunction [36].

We also observed that aspirin treatment was involved in regulating GSH-dependent homeostasis as well as CYP-dependent metabolism in the pancreas and heart of GK rats. Our results showed alterations in the GSH-antioxidant pool as well as the GSH-metabolizing enzymes, which were more pronounced in the mitochondria, indicating increased mitochondrial stress. Studies have shown that mitochondrial GSH is the main line of defense for maintaining the appropriate mitochondrial redox environment [60]. Aspirin treatment improved the redox homeostasis, though the effects were differential in the pancreas and heart. Similarly, CYP 2E1 and CYP 3A4 activities were increased in GK diabetic rats, suggesting that ROS mediated oxidative and metabolic stress. CYP 2E1 activity decreased significantly after aspirin treatment. CYP 3A4 activity decreased slightly after aspirin treatment in the pancreas but increased further in the heart. This may be due to differential isoenzyme-specific metabolism of aspirin in the pancreas and heart. These results have confirmed our previous observation of altered drug metabolism and a marked induction in CYP-dependent metabolism in different tissues in diabetic rats [26,27,28,30]. Aspirin treatment thus appears to be beneficial in improving the drug-metabolizing abilities in these tissues. Researchers have shown that aspirin treatment induced the expression of aryl hydrocarbon receptors, a cytosolic transcription factor, which activated the phase I (cytochrome P450) and phase II (GSTs) detoxification enzymes [61].

Mitochondria produce ROS as a by-product of normal cell metabolism through oxidative phosphorylation. Physiological levels of ROS enhance glucose sensitivity and insulin secretion. Altered metabolism, however, can lead to the rapid overproduction of ROS due to the increased production of oxidants such as superoxides, peroxyl and hydroxyl radicals, as well as peroxides, causing damage to mitochondrial proteins [62]. Mitochondrial GSH and GSH-metabolizing enzymes, in addition to SOD, form the physiological cellular defense system in the control of ROS. However, an imbalance between ROS production and cellular antioxidant defenses can lead to persistent oxidative stress [63]. This could alter mitochondrial function, leading to beta-cell dysfunction and thereby cause insulin resistance [54]. Along with excessive ROS production, mitochondrial dysfunction is also characterized by the disturbance of calcium homeostasis and the mitochondrial permeability transition (MPT) pore opening, which can lead to swelling of the mitochondria, collapse of the membrane potential and ultimately cell death [2]. Increases in mitochondrial ROS production and alterations in mitochondrial structure and functions have been shown in most vital organs and tissues of diabetic animals and patients [2,64]. Studies have suggested mitochondrial dysfunction to be a thread running deep in T2D, not only in organs that are primary drivers of the disease but also in those impacted secondarily [38]. Mitochondrial dysfunction and oxidative stress have been strongly linked to cardiovascular disorders, especially in diabetic patients [8,65]. Researchers have shown that hyperglycemia induces metabolic changes in pancreatic beta cells that markedly reduce both oxidative and glycolytic glucose metabolism and thus ATP synthesis [66]. In addition, impaired mitochondrial energy metabolism has been shown to be accompanied by reduced insulin secretion [64]. Under normal physiological conditions, cardiac ATP production is derived from the mitochondrial oxidation of carbohydrates and fatty acids (FAs), with fatty acids being the preferred substrate. The diabetic heart, however, relies on mitochondrial fatty acid oxidation for ATP synthesis, and this reliance has detrimental consequences, including impaired mitochondrial respiratory function [65]. Furthermore, since the diabetic heart is known to have a diminished mitochondrial antioxidant capacity, minor alterations in mitochondrial structure or function induced by increased ROS production are associated with major alterations in the functioning of the heart muscle [67]. Our results have shown compromised mitochondrial respiratory function with altered enzyme activities of the respiratory complexes in the heart and pancreas of GK rats. These results confirm our previous observation of alterations in mitochondrial activities in type 1 and type 2 diabetic rat models [27,28,29,30]. Studies have reported an increase in the expression of genes that are significant in increasing the electron transport chain pathway and ATP levels by activating the complexes required for cell survival and mitochondrial respiration [61]. This study has also reported increases in HO1 and Nrf2 genes, which serve as an essential antioxidant defense. In our present study, aspirin treatment showed beneficial effects by recovering redox homeostasis, altering mitochondrial functions in favor of energy metabolism, with increased ATP production and improved insulin signaling, as indicated by an increase in the activity of GDH, a mitochondrial Krebs cycle enzyme, which was inhibited in the pancreas and heart of GK animals. Studies have shown that the activation of GDH increased the level of TCA cycle intermediates and restored ATP levels, thus protecting pancreatic cells from high-glucose-induced apoptosis [68].

## 5. Conclusions

Mitochondria play a crucial role in energy metabolism, signaling and cell death in metabolic disorders such as diabetes, causing cardiac dysfunction. The main source of endogenous ROS is mitochondrial oxidative phosphorylation. NOX and cytochrome P450 enzymes are other endogenous sources. GSH is the main line of defense for maintaining the appropriate mitochondrial redox environment. Our study in the pancreas and heart of type 2 diabetic GK rats showed alterations in the GSH-antioxidant pool, as well as the GSH-metabolizing enzymes, and also increased ROS production and lipid peroxidation, indicating increased mitochondrial stress, which in turn could cause mitochondrial dysfunction. Aspirin treatment regulated the intracellular ROS levels via the elevation of the GSH pool and activation of antioxidant enzymes, such as SOD, which quenches superoxide anion radicals, and glutathione peroxidase, which regulates hydrogen and lipid peroxide levels. The recovery of redox homeostasis by aspirin treatment thus could be a possible mechanism in the improvement of oxidative-stress-dependent alterations in mitochondrial functions which in turn facilitates improved energy metabolism in these animals. Thus, the maintenance of mitochondrial integrity and biogenesis by means of the regulation of redox homeostasis could be a promising therapeutic strategy in controlling diabetes and related cardiovascular complications.

## 6. Limitations

In our present study, we have shown that aspirin treatment improved mitochondrial energy metabolism and redox homeostasis in GK diabetic rats, which may have implications in cellular signaling and insulin responses, as demonstrated in our earlier studies. However, a limitation of our study might be that we could use an interventional approach and further elucidate the complete mitochondrial respiratory functions via oxygen utilization, for example, using the Seahorse assay, as well as mitochondrial dynamics using fission and fusion markers. Furthermore, the alterations in the activities/concentrations of the respiratory enzyme complexes could be confirmed by Western blot analysis. Nonetheless, our data are in agreement with our previous studies using GK and other diabetic rat models. Moreover, the direct effects of aspirin on cardiomyocytes and pancreatic beta cells in vitro need to be studied.

## Data Availability

The data used to support the findings of this study are included in the manuscript.
